# Progress in Research on Co-Packaged Optics

**DOI:** 10.3390/mi15101211

**Published:** 2024-09-29

**Authors:** Wenchao Tian, Huahua Hou, Haojie Dang, Xinxin Cao, Dexin Li, Si Chen, Bingxu Ma

**Affiliations:** 1 School of Electro-Mechanical Engineering, Xidian University, Xi’an 710071, China; 23041212927@stu.xidian.edu.cn (H.H.); d2100710345@outlook.com (H.D.); 23041212584@stu.xidian.edu.cn (X.C.); 22041212901@stu.xidian.edu.cn (D.L.); 2 State Key Laboratory of Electromechanical Integrated Manufacturing of High-Performance Electronic Equipments, Xi’an 710071, China; 3 The Fifth Electronics Research Institute of Ministry of Industry and Information Technology, Guangzhou 510000, China; chensiceprei@yeah.net; 4 The Science and Technology on Reliability Physics and Application of Electronic Component Laboratory, China Electronic Product Reliability and Environmental Testing Research Institute, Guangzhou 510000, China; mbxscut@163.com

**Keywords:** co-packaged optics, optoelectronic integration, advanced packaging, silicon photonics integration

## Abstract

In the 5G era, the demand for high-bandwidth computing, transmission, and storage has led to the development of optoelectronic interconnect technology. This technology has evolved from traditional board-edge optical modules to smaller and more integrated solutions. Co-packaged optics (CPO) has evolved as a solution to meet the growing demand for data. Compared to typical optoelectronic connectivity technology, CPO presents distinct benefits in terms of bandwidth, size, weight, and power consumption. This study presents an overview of CPO, highlighting its fundamental principles, advantages, and distinctive features. Additionally, it examines the current research progress of two distinct approaches utilizing Vertical-Cavity Surface-Emitting Laser (VCSEL) and silicon photonics integration technology. Additionally, it provides a concise overview of the many application situations of CPO. Expanding on this, the analysis focuses on the CPO using 2D, 2.5D, and 3D packaging techniques. Lastly, taking into account the present technological environment, the scientific obstacles encountered by CPO are analyzed, and its future progress is predicted.

## 1. Introduction

Data-intensive applications such as artificial intelligence, machine learning, high-resolution video streaming, and virtual reality have grown rapidly in recent years. The exponential growth of data traffic from hyperscale data centers and cloud service providers has led to a substantial rise in the need for network infrastructure bandwidth. As a result, the total bandwidth of switch systems and Ethernet fiber has been doubling every two to three years. The exponential growth in bandwidth has resulted in a proportional rise in power usage. Conventional data centers are constructed with predetermined power limits and predictions of energy consumption. As a result, the bandwidth must be adjusted within a narrow power range, or else expensive improvements to the infrastructure become necessary. The increasing need for data center bandwidth and the accompanying power consumption difficulties are fueling the requirement for inventive solutions [1]. Conventional optoelectronic integration involves the use of optical modules placed at the edges of the panels. These modules can be either plug-in optic modules or source optical cables, which are integrated onto the printed circuit board. This method has reached a higher level of development, has been developed with a focus on modularity, and is straightforward to maintain. Nevertheless, this approach employs lengthier electrical connecting lines, which exhibit evident parasitic effects, and encounters a signal integrity issue. Simultaneously, the module has a bigger physical size, a low density of interconnections, and consumes power over several channels [2,3,4].

**Figure 1:**
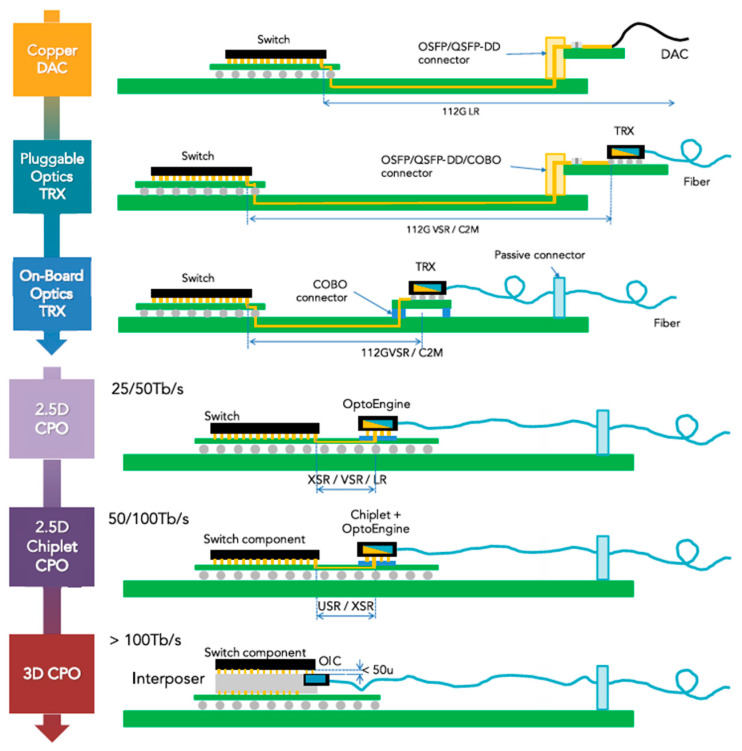
Switch ASIC with pluggable optics versus co-packaged optics [2].

Co-packaged optics with Application Specific Integrated Circuits (ASICs) via low-loss electrical channels are considered the next step in integrating density, cost-effectiveness, and energy efficiency. CPO is a new type of optoelectronic integration technology. Figure 1 shows that the photoelectric interconnection is developing from the traditional board-side optical module to the direction of higher integration and smaller size. In today’s approach, the switch ASIC sits on top of the first-stage package, which in turn is connected to the motherboard. Pluggable optics are placed on the edge of the motherboard and driven by wires that are usually a few inches long. Based on advanced packaging technology, the co-packaging optical side heterogeneously integrates the optical transceiver module and the ASIC chip that controls the operation in a single package, which shortens the wiring distance between the chip and the module, and finally packages the optical engine and the electrical switch chip into a single chip to form a microsystem with certain functions [5,6,7]. This overcomes the Ball Grid Arrays (BGA) and Land Grid Arrays (LGA) pin density limitations and drives the transceiver with shorter wires to significantly reduce channel losses and thus reduce energy consumption. Advanced packaging technology is a process technology that utilizes advanced design concepts and integrated process technologies such as through-silicon via (TSV), the Re-Distribution Layer (RDL), flip chip, bumping, and wire bonding to restructure chips at the package level, effectively improving functional density [8,9]. Based on Broadcom data, the power consumption of pluggable optical modules varies from 15 pJ/bit to 20 pJ/bit. However, the power consumption of CPO systems can be decreased by more than 50% to a range of 5 pJ/bit to 10 pJ/bit [10]. The simulation findings demonstrate a decrease in time of up to 40% when using the all-to-all communication mode. By implementing CPO technology in switches and servers, it is possible to increase network capacity by 2-fold while simultaneously decreasing the number of switches by 64% [11].

## 2. Research Status and Application Prospects of CPO

### 2.1. Research Status of CPO

Since 2020, the industry has begun to establish a consensus around the establishment of CPO standards. Technological and industrial progress is strongly associated with the development of standards, and the United States, China, and Europe are at the forefront of standardization initiatives. Organizations including the Optical Internetworking Forum (OIF), Consortium for On-Board Optics (COBO), The International Photonics & Electronics Committee (IPEC), and the China Computer Interconnection Technology Alliance (CCITA) have made substantial progress in implementing CPO standards. Switch and optical module firms are currently the main promoters of CPO technology. Intel, Broadcom, Marvell and other leading companies in the industry have launched a number of products based on CPO technology. Meanwhile, other firms are aggressively adopting CPO-related technologies and products, and campaigning for the standardization of CPO technology. Meta and Microsoft have formed the CPO Collaboration, a platform aimed at attracting top companies from different industries to collaborate on developing CPO standards and products. The present focus of the proposed CPO optical engine technology solutions is on utilizing Vertical-Cavity Surface-Emitting Laser (VCSEL) solutions and Silicone Optical Integration Program.

Over the past few years, there has been a continuous release of silicon photonics CPO prototypes. Intel is dedicated to conducting research and development on pluggable optical modules and microring modulator technology. Since 2020, Intel has been implementing these technologies in the field of CPO. They utilize their distinctive silicon photonics process platform to create a CPO system that relies on a microring modulator. Intel introduced its inaugural CPO prototype at Optical Fiber Communication Conference & Exposition and the National Fiber Optic Engineers Conference (OFC) 2020 [12]. The switch consists of a 1.6 Tbit/s silicon photonics engine integrated with a 12.8 Tbit/s programmable Ethernet switch, with copper coolers on the left and right sides, connected to the package by heat pipes, and it is architecturally designed with heat dissipation in mind.

In 2021, Intel collaborated with Ayar Labs to combine the Stratix10 FPGA chip and five optical IO TeraPHY chips on a 16-layer organic substrate. This resulted in a multi-chip package with a bandwidth of 8 Tbps, which is suitable for usage in switch devices [13]. In 2022, Intel announced a new partnership with Ayar Labs. They utilized FPGAs and silicon photonics processors to create an optical input/output (IO) link. This collaboration successfully tested signal connectivity with a bandwidth of 5.12 Tbps, marking the first time this level of performance has been achieved [14]. During the 2024 IEEE International Solid-State Circuits Conference (ISSCC), Intel unveiled its most recent advancement in CPO technology. This breakthrough enables signal transmission at a rate of 4 × 64 Gb/s while maintaining a remarkably low system power consumption of only 1.3 pJ/bit [15]. Furthermore, Intel has created a distinctive pluggable optical connector that can examine photonic integrated circuits (PICs) prior to packaging, resulting in improved yields and establishing the groundwork for large-scale manufacture of CPOs [16].

At the 2022 OFC conference, Broadcom introduced its inaugural CPO switch, which combines a 25.6 Tbps Tomahawk 4 switch chip with optics [17]. Broadcom introduced the Tomahawk StrataXGS 5 (Broadcom, Irvine, CA, USA), its newest switch product, in 2023. This switch has a switching capacity of 51.2 Tbps, power consumption of only 5.5 W, and a rate of 800 Gbps. The optoelectronic co-packaging method significantly reduces the power needed for optical communication by over 50%. During OFC 2024, Broadcom presented a switch system with a capacity of 51.2 T, equipped with CPO technology and including eight 6.4 T FR4 light engines. Each optical engine consists of 64 channels of photonic integrated circuits (PIC) and electrical integrated circuits (EIC). The Driver and Trans-impedance amplifier (TIA) chips are manufactured using Complementary Metal Oxide Semiconductor (CMOS) technologies. The signal rate of a single channel is 100 gigabits per second (Gbps), and the light engine is utilized in fan-out wafer-level packaging (FOWLP) [10,18].

Ranovus’ technology emphasizes the utilization of quantum dot lasers, microring modulators, and other related components. Ranovus introduced the Odin Brand Simulation-Driven CPO 2.0 architecture at OFC 2021. This architecture was collaboratively developed by Ranovus, IBM, TE, and Senko. CPO 2.0 surpasses CPO 1.0 by achieving a 40% decrease in power consumption and cost savings through the elimination of the retiming function and the implementation of an IC-effective single-die solution, resulting in a lower overall size [19]. Ranovus presented a device at OFC 2023 that combines an 800 G direct-drive silicon photonics engine with AMD’s FPGA chip [20]. Cisco showcased its 25.6 T switch prototype based on CPO technology at OFC 2023. The system has eight 3.2 T silicon photonic engines, each equipped with eight 400G-FR4 silicon photonic chips. Each individual optical engine operates at a single-channel throughput of 100 Gbps [21]. Table 1 is a comparison of the CPO results of the above companies based on silicon photonics integration technology.

Vertical-Cavity Surface-Emitting Lasers offer clear benefits in terms of cost and power usage for ultra-short-haul transmission [22]. The primary organizations now involved in the research and advancement of VCSEL CPOs are IBM, HP, Fujitsu, and Furukawa.

In 2022, IBM Research and Coherent joined together to work on the MOTION project [23], which focuses on creating compact optical modules that integrate several wavelengths on a single chip. The module utilizes a chip with dimensions of 1.64 mm × 4.64 mm. It does not incorporate the retiming function within the electrical chip, allowing it to effectively serve low-latency application scenarios. The electrical chip, VCSEL, and power delivery (PD) chips are affixed to a glass substrate using a flip-mounting technique. This module is characterized by its remarkable affordability and minimal energy usage. At its maximum speed, the MOTION transceiver consumes 4 pJ/bit, taking into account the electrical connectors on both ends. This is significantly lower, five times to be exact, than the 800 G OSFP (FR4) module, which is considered the most advanced technology now available.

HP’s 4-channel CPO system, developed in 2020, includes five wavelengths of VCSEL lasers at 990/1015/1040/1065/1090 nm. The 3 dB bandwidth of the five wavelengths is greater than 18 GHz and can support 56 Gbps PAM4 signals [24].

Fujitsu’s VCSEL CPO system, announced in 2022, utilizes a 16-channel VCSEL and PD array. To enable coupling with multi-core fiber (MCF), the VCSEL and PD are arranged in an arc, with a 40 μm distance between adjacent channels. The VCSEL emits single-mode 1060 nm output, and the driver and TIA chips are positioned on the back of the interposer [25].

Furukawa’s VCSEL CPO solution utilizes two sets of 4-channel VCSEL and PD arrays. To minimize the length of the electrical signal interconnection, the driver and TIA chips are positioned on opposite sides of the VCSEL and PD, respectively. Both optical and electrical chips are directly affixed to the substrate and connected through wire bonding [26].

At present, VCSEL CPO is still in the research and development stage, and its main challenge lies in packaging. The packaging solutions across different companies typically involve connecting the optical engine to the printed circuit board (PCB) using a Land Grid Array (LGA) package. It is essential to position the driver and TIA as close as possible to the VCSEL and PD. Moreover, the system’s reliability and maintenance are put to the test with the integration of multi-channel VCSELs and PDs [27].

With the increase in VCSEL modulation rate, the reliability of the chip decreases. At 56 GBaud, there is no stable and reliable large-scale integrated VCSEL array. Therefore, there are relatively few studies on multi-channel parallel optical interconnection based on VCSEL array schemes. In recent years, silicon photonics integration technology has become the main solution for CPO optical engines. Silicon photonics do not require hermetically sealed packaging, CMOS compatibility makes it easier to integrate with electrical chips, and both silicon photonics modulators and detectors can support speeds above 56 GBaud [28,29,30]. Table 2 shows a comparison of the VCSEL-based CPO results of the above companies.

### 2.2. Application of CPO

#### 2.2.1. Military Field

The sensor using CPO technology has the advantages of high detection sensitivity, small size, strong battlefield adaptability, and low cost. They can be used in the next generation of intelligent detection equipment to optimize and upgrade combat performance. Lidar has become an indispensable key sensor for military reconnaissance and surveillance. Due to the large refractive index differences of the SOI materials used in silicon photonic chips, the size of these devices can be significantly reduced. Some countries have developed all-solid-state lidars based on silicon-based optical phased array chips, which are expected to provide key technical support for the next generation of military lidars [31].

Ayar Labs and Lockheed Martin have integrated TeraPHY co-packaged optical I/O chips with radio frequency integrated circuits (RFICs) to build advanced packages with these capabilities. Sensors, switches, and processing elements can utilize this optical I/O to significantly reduce power consumption and increase bandwidth. Ayar Labs and Lockheed Martin compared co-packaged optical I/O with existing state-of-the-art mid-plate optical transceivers, and the results showed that the optoelectronic co-packaged devices have lower power consumption, smaller area, lower error rates, and higher performance [32].

#### 2.2.2. Data Center

Co-packaged optics technology can achieve higher data density and faster data transmission speed, and can be applied to high-speed network switching, server interconnection, and distributed storage. In 2023, the IBM Thomas J. Watson Research Center explored the advantages of using co-packaged optics to build low-diameter, large-scale high-performance computing (HPC) and data center networks [33]. Comparing the baseline architecture to a system with co-packaged optics integrated on switch nodes and accelerator chips, the researchers found that with 256 nodes and a message size of at least 128 KiB, the average server throughput increased by more than 90%. Switches with co-packaged optics simplify network topologies, with twice the number of servers per leaf switch and four times the high bisecting bandwidth when using 41% of switches.

#### 2.2.3. Cloud Computing

The unique advantages of CPO technology have brought significant performance gains and cost savings to cloud computing. First of all, with the continuous growth of cloud computing business, the requirements for data transmission speed and efficiency are also increasing. CPO technology significantly improves data transmission speed and quality by reducing signal attenuation and shortening the transmission path. In addition, the co-packaged optics technology reduces the overall power consumption of the system by optimizing the layout and connection of optoelectronic devices, which helps to achieve green cloud computing. CPO technology enables cloud computing platforms to process data more efficiently and support more concurrent users and services. At the same time, due to the high scalability of CPO technology, the cloud computing platform can be flexibly expanded and upgraded according to business needs.

## 3. Typical Package Form of CPO

According to its physical structure, CPO can be divided into three forms: CPO in a 2D package, CPO in a 2.5D package, and CPO in a 3D package.

### 3.1. CPO Based on 2D Packaging

Co-packaged optics technology based on 2D packaging involves placing a PIC and an EIC side by side on a substrate or PCB, and establishing interconnections through leads or substrate wiring. The advantages of 2D packaging include ease of packaging and high flexibility. Both EIC and PIC can be manufactured separately using different materials and processes. Three technical paths have been developed based on 2D packaging, depending on the interconnection methods of chips and substrates: CPO based on wire bonding, CPO based on flip form, and CPO based on fan-out wafer-level packaging technology [34].

#### 3.1.1. CPO Based on Wire Bonding

**Figure 2:**
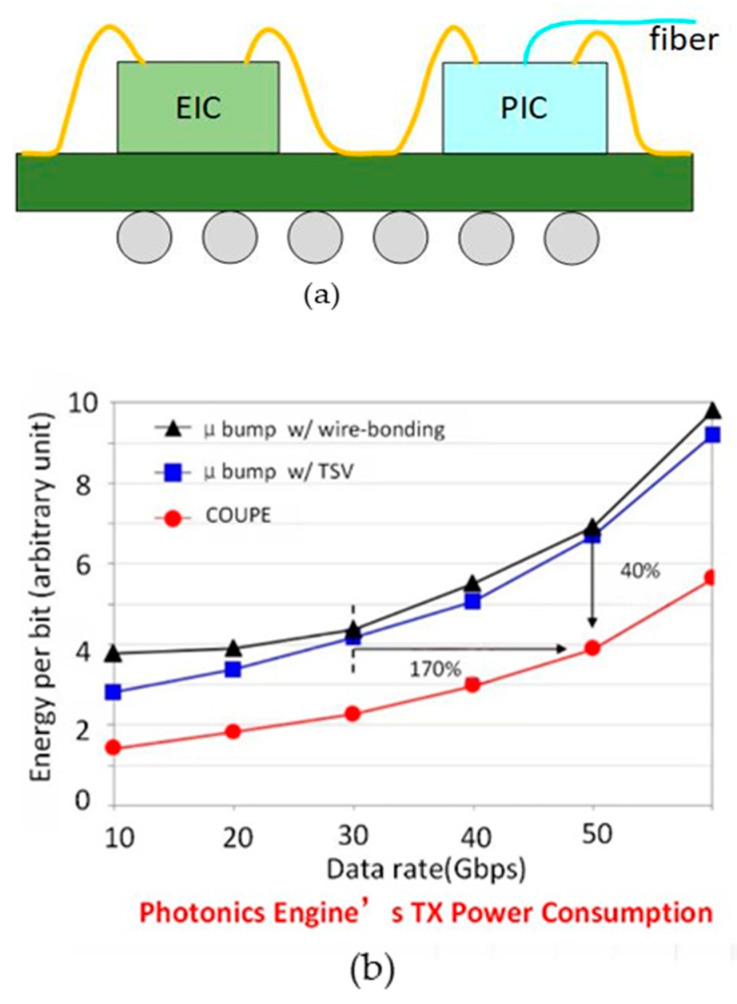
(**a**) The package structure of COUPE technology; (**b**) performance comparison of 3D micro-bump technology with wire bonding and 3D micro-bump technology with TSV at different rates [35]. Copyright © 2021, IEEE.

In 2021, TSMC introduced the Compact Universal Photonic Engine (COUPE) technology to enhance bandwidth and energy efficiency. This technology integrates EIC and PIC on the same substrate and connects them through wire bonding, as illustrated in Figure 2. The COUPE scheme boosts the signal rate by 70% compared to the microbump + TSV approach [35].

However, the connection point between the lead and the chip can move or fatigue due to factors such as thermal stress, resulting in package failure. In addition, wire bonding often requires a relatively high arc height to allow for cyclic connections between chips and substrates or between chips and chips, which is not conducive to miniaturized designs.

#### 3.1.2. Flip-Based CPO Technology

**Figure 3:**
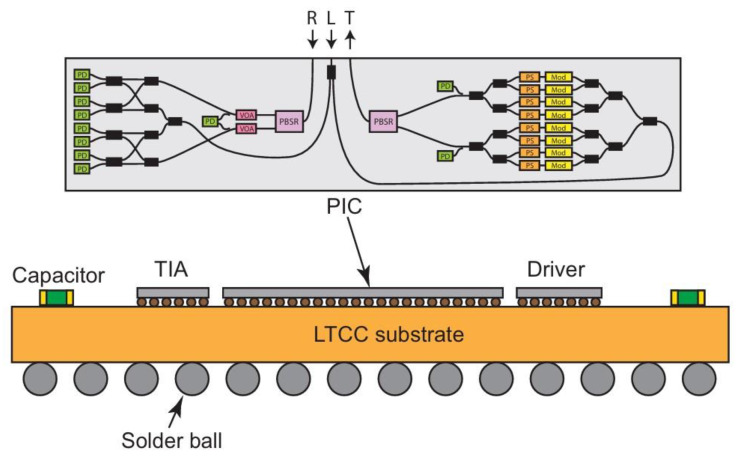
Schematic diagram of CPO technology based on ceramic substrate [36]. Reprinted with permission from © Optical Society of America.

Acacia designed a transceiver using CPO technology to flip-weld the PIC, Driver, and TIA to an 11-layer Low Temperature Co-fired Ceramic (LTCC) substrate. This approach enables the optoelectronic co-packaging of ASIC chips and optoelectronic integrated circuits, as illustrated in Figure 3. The signals are interconnected through the internal wiring of the ceramic substrate, which offers a short path and excellent electrical performance. Compared to wiring on the PCB, the transmission loss is significantly reduced, and the structure is more compact, ensuring the product’s optimal electrical performance, heat dissipation, and stability [36].

This approach saves costs, reduces size, and enhances heat dissipation and bandwidth. The substrate is made of low-temperature co-fired ceramics, which are 10 times less expensive than traditional high-temperature co-fired ceramic (HTCC) packages. The Ball Grid Array Package (BGA) substrate supports higher bandwidths and utilizes metals—copper and gold—with lower RF losses. The BGA package excels in heat dissipation because the back of the mold is directly attached to the lid. Moreover, the BGA package accommodates a large number of electrical connections. Ceramic substrates can be utilized for multi-layer wiring, but the wiring line width and line spacing are broad, making it challenging to fan out I/O when the pin density is high. In terms of cost, ceramic substrates are significantly more expensive compared to organic substrates and silicon substrates.

#### 3.1.3. CPO Based on FOWLP

**Figure 4:**
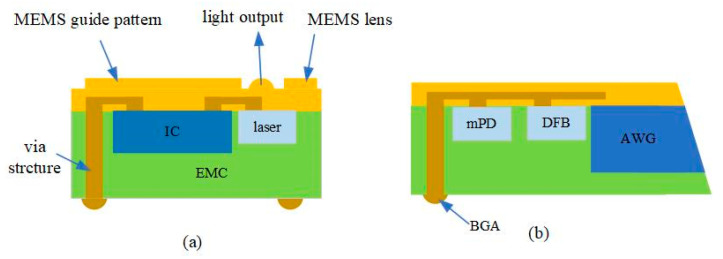
The cross-sections of the hybrid optical package based on FOWLP platform for (**a**) multi-mode and (**b**) single-mode applications. The direction of light is vertical in the multi-mode package and lateral in the single-mode optical package [37]. Reprinted with permission from © Optical Society of America.

Fan-out wafer-level packaging, which is widely used in silicon semiconductor systems, is an integrated hybrid optical package that has the advantages of small size, high performance, and can be used for on-board/co-packaged optical interconnects. Korea’s Lipac reported a new hybrid optoelectronic packaging technology based on fan-out wafer-level packaging (FOWLP). As shown in Figure 4, the electronic chip and the optical chip are embedded in an epoxy molding compound (EMC) substrate, and the optical interconnection is realized through the RDL layer, which eliminates the use of wire bonding or bumps, and therefore shortens the interconnect wires [37].

**Figure 5:**
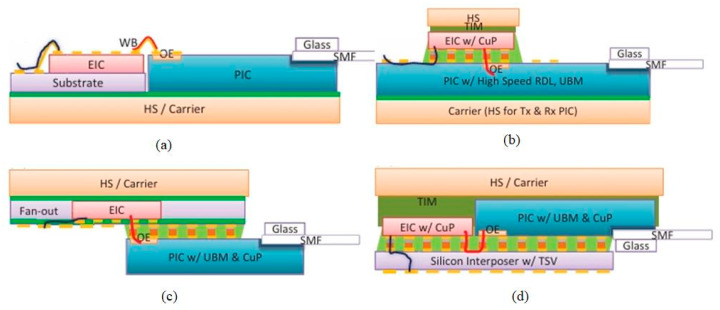
Optical engine integration options using our PIC-EIC chipsets (**a**) wire bonding, (**b**) EIC on PIC, (**c**) PIC on fan-out EIC, and (**d**) 2.5-D integration on silicon interposer [39]. Copyright © 2013, IEEE.

In 2020, Rockley Photonics partnered with Accton, TE, and Molex to demonstrate a 25.6 Tbps OptoASIC switching system, with Rockley’s design addressing signal integrity, integration density, and cost challenges [38]. In 2022, Bruce Chou et al. designed and demonstrated a co-packaged light engine using a chipset designed by Rockley, with a package schematic shown in Figure 5c [39]. In this scheme, the design of a multi-micron silicon PIC combines performance, power efficiency, and manufacturability. Full integration of the optical transmitter and receiver can be achieved with planar integration of an integrated photodetector such as a 100 Gbps integrated photodetector, via pattern-matched V-slots, copper pillar (CuP) collisions, and an electrical absorption modulator (EAM) [28]. Second, the EIC is designed to match the detector/modulator on the PIC, allowing it to optimize the signal integrity of the PIC’s most sensitive interfaces. Finally, FOWLP is applied to the chipset to optimize the integration density. FOWLP can keep EIC costs low by keeping the chip area small, while connecting the Land Grid Array (LGA) to the switch ASIC substrate using a fan-out area, which is performed using a socket. By using socket technology, the light engine will be replaceable within each switch box.

**Figure 6:**
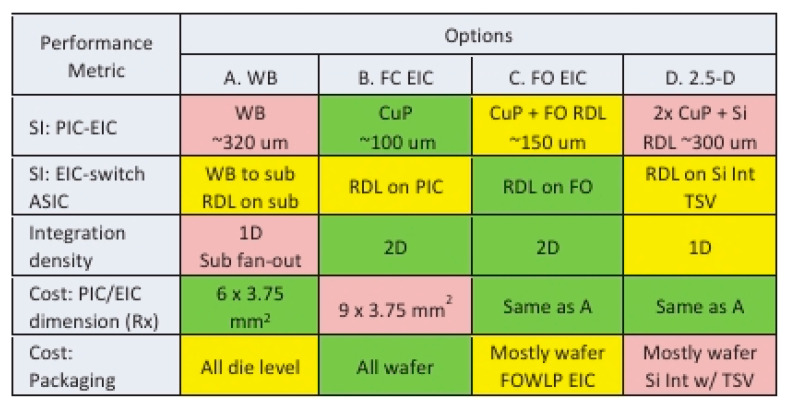
Packaging options by performance metrics [39]. Copyright © 2013, IEEE.

As shown in Figure 5, Bruce Chou’s team compared four package options, namely 2D integration with wire bonding, flip-chip EIC on PIC, flip-chip EIC on PIC, and 2.5-D packaging w/silicon interposer. The main performance comparisons are signal integrity: PIC-EIC, signal integrity: EIC-Switch ASIC substrate, integration density, cost—PIC/EIC dimension, and cost—packaging, and the results are shown in Figure 6, which represents the flip-chip EIC on PIC as the best option among the four packages.

Fan-out wafer-level packaging offers advantages in size and production capacity by facilitating small-size packaging and mass production capabilities through wafer-level packaging. FOWLP also provides higher speed design capabilities by eliminating wire bonding. The FOWLP RDL process requires only a small pad area (as small as 1/10th), resulting in smaller load capacitance and chip area. Decreasing the pad size of the component chip allows for potential optimization of optical and electrical device chips.

The complex process of FOWLP includes wafer reconstruction, molding, rerouting, etc., and each step has a serious impact on package reliability. Wafer reconfiguration technology requires good positioning accuracy, both good adhesive strength and easy peeling, otherwise it will cause the chip to shift. The molding process protects the chip and expands the chip area, and the epoxy molding compound liquefies when heated, encapsulates the die, and solidifies after cooling. There is a large mismatch between the coefficient of thermal expansion of epoxy molding compounds and other materials, and the liquid flow generated during injection molding can also change the grain position, causing wafer warpage and die shift. Rewiring technology is the key technology to achieve the fan-out effect, and Polyimide (PI) is the most commonly used organic material in the rewiring layer. Due to the different coefficients of thermal expansion between metal and polyimide, cracking of the rewiring layer can occur if the strength of the rewiring layer is insufficient during temperature changes.

### 3.2. CPO Based on 2.5D Packaging

In the 2.5D package, both the EIC and PIC are flip to the interposer, and the interposer board is connected to the underlying package substrate or PCB board through the metal interconnection PIC and EIC on the interposer. The size of the 2.5D integrated package is larger than that of the 3D integration, and the signal performance is degraded because the signal has to pass through two bumps. The 2.5D packages have higher interconnection density and lower power consumption. The interposer board has the function of supporting PIC/EIC and realizing PIC/EIC/PCB electrical interconnection. Silicon interposers, glass interposers, and EMIB can be used to integrate electrical chips with silicon photonics chips.

#### 3.2.1. CPO Based on Silicon Interposer

The silicon substrate has a high integrated density, thin thickness, integrated bumps, and a good thermal expansion coefficient of chip materials. These characteristics can reduce the warpage of packaged products and improve reliability. In the case of multi-channel optoelectronic modules, silicon interfacing boards can interconnect the high-density pins of optoelectronic chips to achieve CPO.

**Figure 7:**
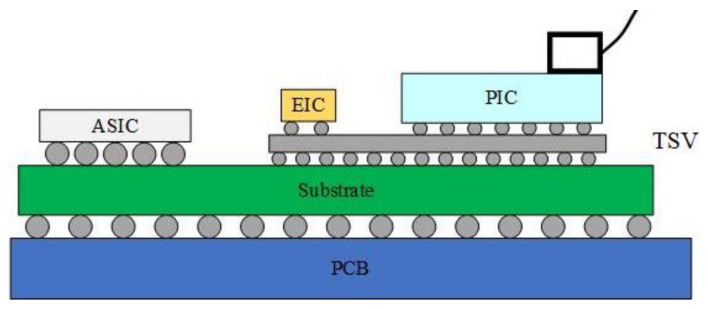
CPO scheme based on silicon interposer [40]. ©2018 by the authors.

In 2021, the Institute of Microelectronics of the Chinese Academy of Sciences developed a high-speed silicon interposer for CPO. The structure of the interposer is illustrated in Figure 7. It features a two-layer RDL structure on the front and a single layer on the back. The optoelectronic chip is mounted on a silicon interlayer to meet the requirements of high-speed electrical interconnection [40].

**Figure 8:**
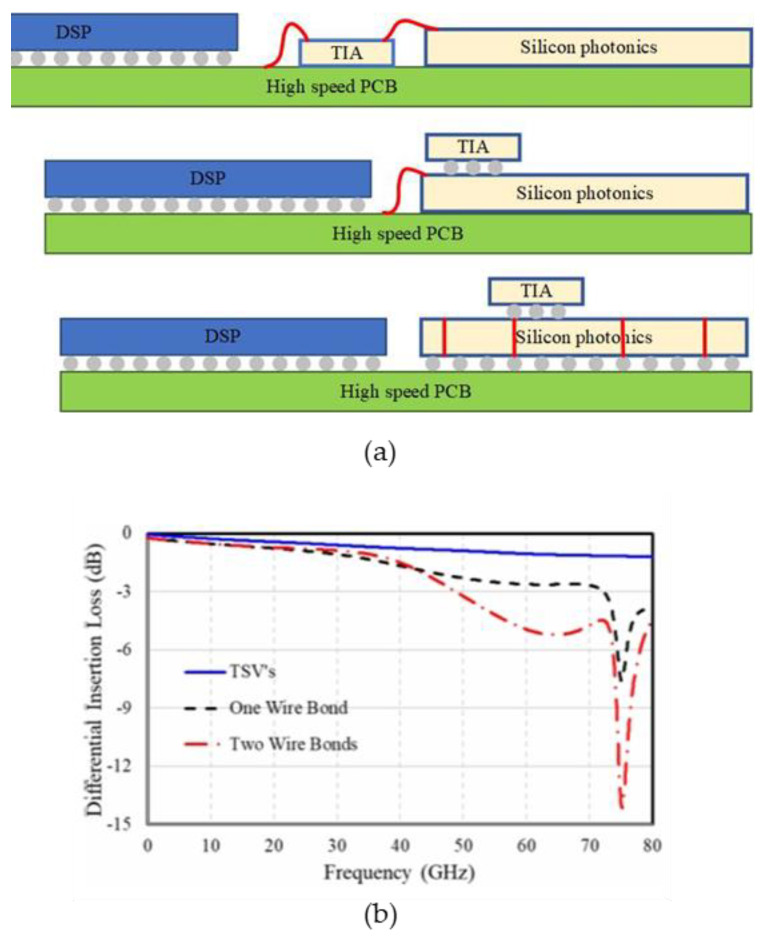
(**a**) Evolution of optoelectronic co-packaging methods. (**b**) Normalized electrical insertion loss as a function of frequency for the three packaging scenarios [41]. ©2023 by the authors.

In 2023, Marvell compared the high-speed performance of 2D packages to 2.5D packages. As shown in Figure 8a below, the first scenario involves placing the digital signal processing chip (DSP), TIA, and silicon photonics chip side by side on the PCB board. The TIA connects the high-speed signal with the PCB board and the silicon photonics chip through wire bonding. The second approach is to flip-chip the TIA chip onto the silicon photonics chip, connecting it to the PCB board through wire bonding, and then linking it with the DSP chip. The third option is to flip-chip the DIA chip onto the silicon photonics chip, with the silicon photonics chip serving as an interposer and connecting the signal with the PCB board through the TSV. The first two options fall under 2D packaging, while the third belongs to 2.5D packaging. The simulation results are depicted in Figure 8b, showing that the 3 dB bandwidth of the first scenario is around 50 GHz, the second around 70 GHz, and the third scenario exhibits superior high-speed performance. Marvell employs a via-last process to handle the silicon photonics chip, followed by utilizing Outsourced Semiconductor Assembly and Testing (OSAT) technology to manage the TSV. Subsequently, the electrical chip is flip-chipped onto the silicon photonics chip, which also serves as an interposer. Compared to the 2D packaging solution based on wire bonding, the high-speed performance of the 2.5D packaging optical engine is superior and not constrained by the signal rate limitations of the wire bonding method. This approach also aids in dissipating heat from the system [41].

As an advanced system integration technology, the silicon-based interposer 2.5D integration technology has developed rapidly in recent years. However, there are two main problems with silicon-based interposers: (1) the high cost, the silicon etching process is used for TSV production, and then the silicon through-silicon vias need to oxidize the insulation layer and hold the thin wafer; (2) the electrical performance is poor, silicon material is a semiconductor material, and when the transmission line transmits the signal, the signal and the substrate material have a strong electromagnetic coupling effect, and the eddy current phenomenon occurs in the substrate, resulting in poor signal integrity.

#### 3.2.2. CPO Based on Glass Interposer

Compared with silicon materials, the Young’s modulus of glass is larger, its hardness is greater, and the thermal expansion coefficient of glass can also be matched with Si and PCB boards to reduce the stress inside the system, so the glass substrate can better solve the warpage problem of large-size chips, so as to improve the yield and reliability of the system.

**Figure 9:**
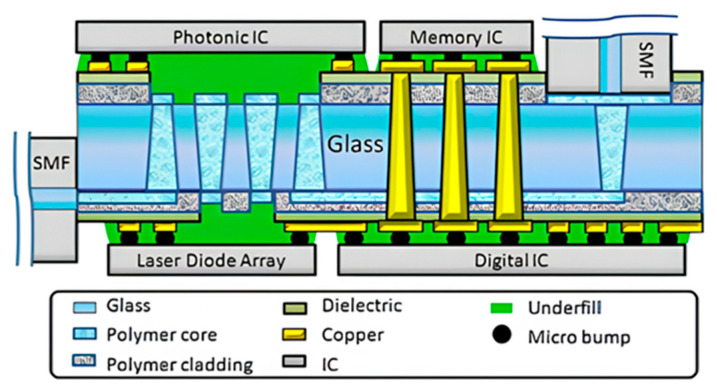
Glass-substrate-based CPO at the Georgia Institute of Technology’s Packaging Research Center [42]. Copyright © 2013, IEEE.

In 2013, the Georgia Institute of Technology’s Packaging Research Center implemented optoelectronic co-packaging on glass substrates. As shown in Figure 9, the system utilizes a 150 μm thick glass carrier board. The PIC, Driver, and TIA components are mounted upside down on the glass carrier board. Light is directed into the waveguide on the back of the carrier board through the organic lens on the carrier board, which is then connected to the optical fiber. The electrical signal is generated by creating a metal through-hole in the glass substrate [42].

**Figure 10:**
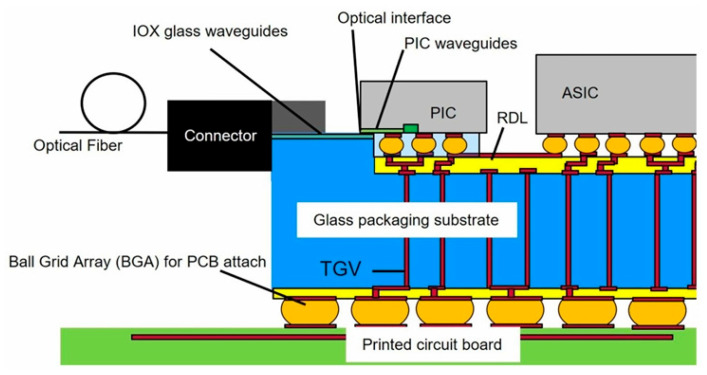
Corning’s glass-based CPO solution [43]. Copyright © 2023, IEEE.

In 2023, Corning experimented with a glass substrate containing optical waveguides to achieve optoelectronic co-packaging. The core components of the glass substrate include through-glass vias (TGV) [43], a SiN waveguide, an adiabatic coupler for coupling PIC to SiO_2_ waveguide, and a fiber connector, as illustrated in Figure 10. An RDL layer is integrated on top of the glass substrate to establish high-speed electrical channels between chiplets. The TGV connector is used for power delivery and grounding. TGV and RDL enable electronic fan-out between the assembled IC and the printed circuit board. Thin-film processing of all high-resolution lines on the same substrate simplifies manufacturing and assembly, potentially reducing overall packaging costs compared to the 2.5D silicon interlayer on organic substrates or embedded multimode interconnect bridging configurations. Furthermore, a glass substrate that integrates a planar ion exchange (IOX) optical waveguide beneath the top surface of the glass provides a strategically positioned optical interface, allowing the assembled PIC to be directly transiently coupled to achieve lower losses.

In recent years, many researchers have devoted themselves to the development of low-cost, small-size, fine-pitch, and non-destructive rapid glass porosity technologies, such as sandblasting, photosensitive glass, plasma etching, focused discharge, laser ablation, etc. [44,45]. However, due to the fragility and chemical inertness of glass materials, there are still many problems with the existing methods. Up to now, the main difficulties in the development of glass through-hole 3D interconnection technology include the following: (1) although the existing methods can achieve TGV, some methods will damage the glass and cause the surface to be unsmooth. (2) TGV requires high-quality filling technology. Unlike TSV, TGV has a relatively large pore size and is mostly through-hole, and the plating time and cost will increase. (3) Compared with silicon materials, due to the smooth surface of glass, the adhesion to commonly used metals (such as Cu) is poor, which makes it easy to cause delamination between the glass substrate and the metal layer, resulting in the curling of the metal layer and even resulting in it falling off. In addition, the heat dissipation capacity of glass is poor, so it is necessary to consider a suitable heat dissipation scheme [46,47].

#### 3.2.3. CPO Based on EMIB

**Figure 11:**
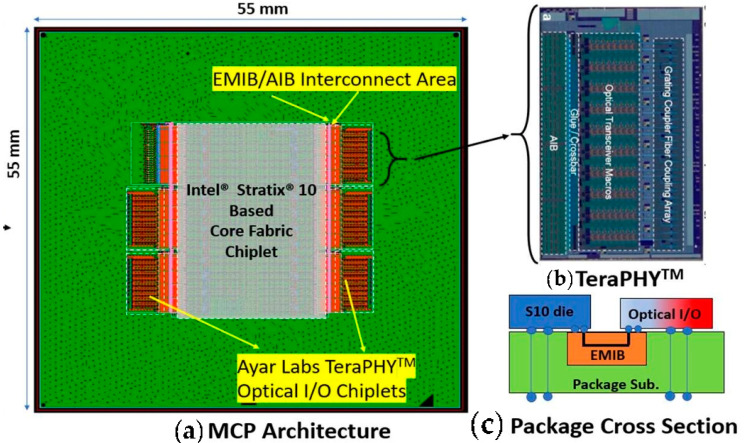
(**a**) Illustration of the MCP with 5 O-chiplets each connected to the FPGA via an EMIB die, (**b**) layout of TeraPHY^TM^ O-chiplet (front view), (**c**) cross-section showing die connectivity through EMIB [14]. Copyright © 2022, IEEE.

The Embedded Multi-die Interconnect Bridge (EMIB) is a 2.5D packaging technology developed by Intel. It involves embedding a thin silicon bridge in an organic substrate and utilizing a multi-layer back-end interconnection process to establish local physical connections, as illustrated in Figure 11. With this technology, Intel and Ayar Labs have successfully integrated the FPGA chip Stratix10 and five optical IO TeraPHY [48] chips on a 16-layer organic substrate measuring 55 mm × 55 mm. The proximity between the optical chip and the FPGA is less than 100 μm, enabling a bandwidth of 8 Tbps. Unlike CPO using a silicon interposer, EMIB technology eliminates signal integrity issues like parasitic capacitance from TSV interposers. Additionally, EMIB requires less space than a silicon interposer, ensuring efficient high-speed, high-density communication between the FPGA and the optoelectronic chip. This advancement results in significant size reduction in the package, leading to improved cost-effectiveness and performance. Compared to existing market products, EMIB technology offers a 5-fold increase in bandwidth, a 5-fold reduction in power consumption, and a 20-fold decrease in latency [14].

Embedded Multi-die Interconnect Bridge technology enables the heterogeneous integration of high-density multichip packages (MCPs) for connections between logic-to-memory and logic-to-electronic transceivers. Unlike traditional 2.5D packaging, EMIB technology does not use TSV, offering advantages such as normal package yield and simple design, and having no additional processes. However, it is difficult for EMIB to keep up with advanced technologies at the beginning of each process release. In addition, there may be differences between the devices at the EMIB end, such as differences in the electrical characteristics and manufacturing processes of the transceiver between Field-Programmable Gate Array (FPGA) and High-Bandwidth Memory (HBM), which can lead to a series of problems, such as uneven heat generation at both ends.

### 3.3. CPO Based on 3D Packaging

Three-dimensional packaging interconnects optoelectronic chips vertically, which can achieve shorter interconnection distances, better high-frequency performance, higher integration, and more compact packaging.

#### 3.3.1. CPO with PIC as Interposer

**Figure 12:**
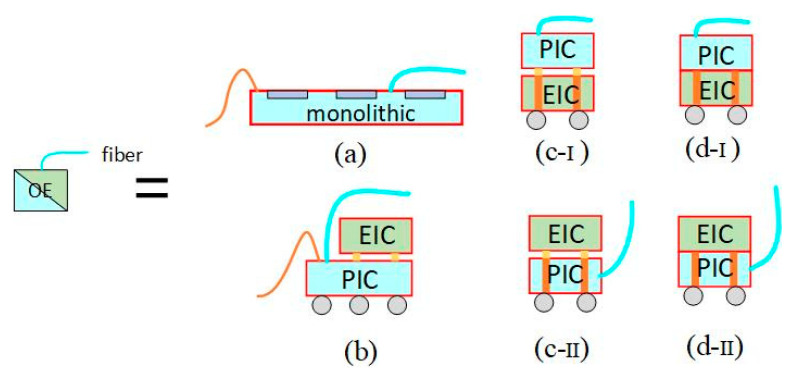
Four types of high-performance OE structures: (**a**) monolithic; (**b**) 3D stacking with TSV; (**c**) 3D stacking w/uBump and TSV; (**c-I**) PIC is an interposer board with TSV, (**c-II**) EIC is an interposer board with TSV, and (**d**) 3D stacking w/ SoIC bond and TSV. (**d-I**) PIC is an interposer board with TSV, and (**d-II**) EIC is an interposer board with TSV [49]. Copyright © 2021, IEEE.

Taiwan Semiconductor Manufacturing Company (TSMC) unveiled its latest silicon photonics packaging roadmap at the Electronic Components and Technology Conference (ECTC) 2023. OE consists of EIC and PIC to facilitate the conversion of electrical signals to optical signals and vice versa. Figure 12 illustrates four high-performance OE structures: monolithic, TSV-free 3D stacking, uBump-free and TSV 3D stacking, SoIC-free bond-free, and TSV 3D stacking. Monolithic OE requires EIC and PIC to utilize the same technology nodes, posing economic challenges as bandwidth requirements increase. The three-dimensional stacking of micro-bumps may not sustain performance expansion over time due to issues like concavity, convexity, or line parasitism. SoIC-based OE, which leverages SoIC bonding technology, minimizes parasitic effects at the EIC-PIC interface, resulting in superior power and performance [49].

**Figure 13:**
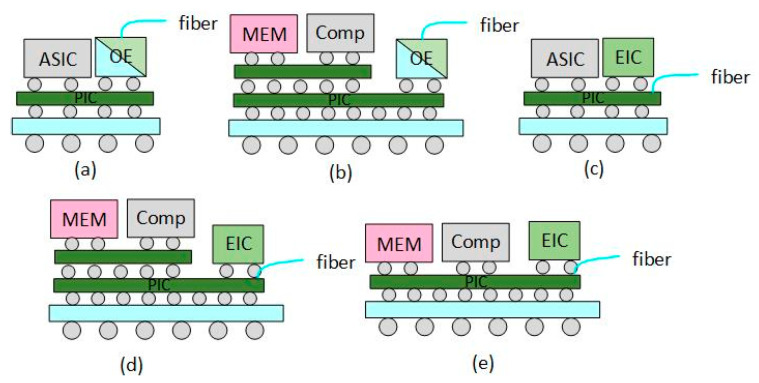
(**a**) has the general form of host ASIC while (**b**) has the specialized Compute-HBM module to replace ASIC. (**c**,**d**), with EIC to replace the stand-alone OE, are simplified versions of (**a**,**b**). (**e**) is the “flattened” version of (**d**). Copyright © 2021, IEEE.

Taiwan Semiconductor Manufacturing Company proposes using a separate PIC as an interposer board to achieve horizontal and vertical electrical interconnection through metal interconnects and TSVs, respectively. TSVs offer several advantages, including reducing package size, shortening interconnect lengths between chips, enabling high-density integration, decreasing transmission delay noise, minimizing chip loss, and enhancing thermal expansion reliability. Figure 13 illustrates five CPO structures utilizing a PIC as an interposer: (a) the ASIC chip and OE chip are positioned on the PIC interposer, (b) the MEM-Comp module and OE module are situated on the PIC, and (c), (d), and (e) involve replacing the OE module with other EICs.

#### 3.3.2. CPO with EIC as Interposer

**Figure 14:**
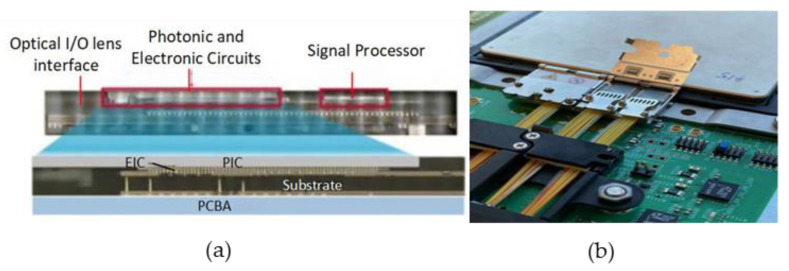
(**a**) High-density CoW components in the Broadcom CPO system; (**b**) pluggable optical connectors developed by Broadcom [51]. Copyright © 2023, IEEE.

In 2022, Broadcom launched a 3D package-based light engine. This innovation involves flipping the PIC above the EIC and interconnecting the EIC and ASIC chips through the substrate to package the 25.6 Tbps Tomahawk 4 switch chip and 4 CPO structure optical engines together to create a switch. Each CPO module supports 3.2 Tbps, and the entire system comprises four CPO modules with a combined bandwidth of 12.8 Tbps. The power consumption of the CPO system can be reduced to 5–10 pJ/bit, compared to 15 to 20 pJ/bit for pluggable optical modules. Broadcom said that the use of CPO structure can save 40% power consumption and 40% cost per bit. The cross-section of the entire chip is illustrated in Figure 14a below [50].

The manufacturing process of 3D packaging is complex, the cost is high, and the material and manufacturing accuracy of the package body are high. TSV interconnect is the core technology of 3D integration. The TSV manufacturing process includes the following: through-hole fabrication; deposition of insulation, barrier, and seed layers; copper filling; removal of excess metal by chemical–mechanical polishing; wafer thinning; wafer bonding, etc. Each step of the process has a high technical difficulty, and these processes directly determine the performance indicators of TSV, which leads to the reliability problem of 3D packaging.

#### 3.3.3. CPO Based on Organic Substrates

The chip-embedding technology of organic substrates can not only improve the integration of chips, but also improve the signal transmission performance, providing an effective solution for achieving high integration and high-performance packaging.

**Figure 15:**
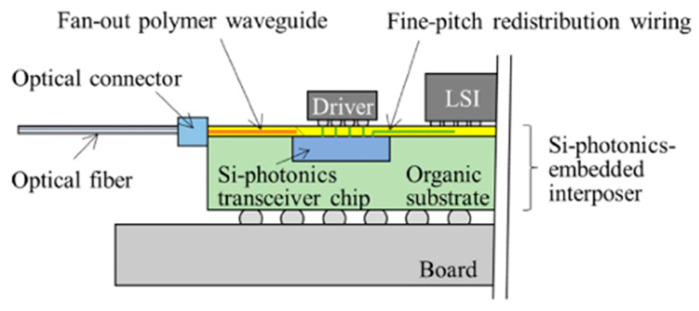
Schematic diagram of silicon photonic intercalation intermediates [52]. Copyright © The Japan Institute of Electronics Packaging.

In 2022, the Japan Optoelectronic Technology Research Association proposed silicon photonic intercalation intermediates as a new packaging platform to realize optoelectronic co-packaging. A schematic diagram of the structure is shown in Figure 15. Silicon photonics transceiver chips are separated from the fiber optic connection and integrated into an organic substrate. Single-mode polymer optical waveguides were fabricated on substrates and embedded silicon photonics chips. The polymer waveguide is connected between the optical I/O interface on the silicon photonics chip and the optical connector at the edge of the intermediate substrate. Single-mode fiber is connected to a polymer waveguide via a connector. In terms of electrical connection, a redistribution layer is fabricated directly on top of the embedded silicon photonics chip and the middle layer. RDL connects embedded transceiver chips to host large-scale integrated circuits (LSIs) [52]. In this structure, the size of the silicon photonic transceiver chip can be minimized.

The manufacturing cost of organic substrates is low, and the manufacturing process is difficult. However, the coefficient of thermal expansion (CTE) of the organic interlayer board is large and the dimensional stability is poor, which leads to the limitation of the interconnection line width and I/O density. In addition, the thermal conductivity of organic matter is low, the heat dissipation performance is poor, and the elastic modulus is small, which is easy to warp during the manufacturing process.

Co-packaged optics is currently in the early stage of industrialization, and each packaging technology has its pros and cons, so it is necessary to constantly search for the most reliable solution. The advantages of 2D packaging are ease of packaging and high flexibility. However, as the bandwidth demand continues to increase, the influence of parasitic parameters on leads and bumps cannot be eliminated, and the interconnection density cannot continue to increase, which will limit the further improvement in system performance. Currently, 2.5D and 3D packaging are the most promising solutions to achieve high-speed optoelectronic co-packaging. Compared with the 2.5D package, the parasitic parameters of the electrical interconnection between EIC and PIC in the 3D package are further reduced, and the bonding spacing is further reduced, which is conducive to the realization of higher bandwidth signal interconnection. This is the current hotspot and trend of CPO technology research.

## 4. Challenges

CPO is the best packaging solution in the long run to achieve high integration, low power consumption, low cost, and future ultra-high-speed module applications. While CPOs have significant potential advantages, they also face challenges with their system integration.

### 4.1. Encapsulation Technology

**Figure 16:**
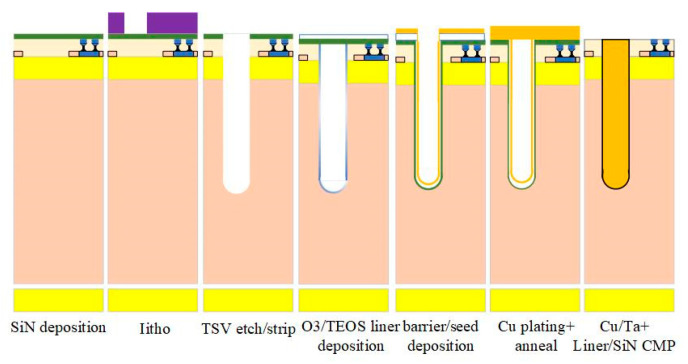
Main preparation process steps of TSV based on silicon interposer [54]. Copyright © 2018, IEEE.

Co-packaged optics involves key technologies in advanced packaging such as TSV, TGV, multilayer high-density interconnect substrates, bumping, and die stacking, each with its own advantages and disadvantages. For example, TGV through-hole technology may damage the glass and cause the surface to be unsmooth. Most TGV processing methods are inefficient and cannot be mass-produced. The plating cost and time of TGV structure are slightly higher than that of TSV, and the adhesion of the glass substrate surface is poor, which can easily lead to the abnormality of RDL metal layer. The fragility and chemical inertness of glass itself make process development challenging. As shown in Figure 16, fluoride $SF_{6}$ and $C_{4}F_{8}$ are used to etch silicon at room temperature. After the etching is completed, the TSV through-hole plating is filled with materials such as copper, silver, and tungsten. To ensure the reliability of TSV, it is necessary to control the plating filling process. Generally, the filling rate of the bottom of the hole needs to be higher than the filling rate of the hole opening and the hole sidewall position to prevent holes from forming inside the hole after filling. Simultaneously, the thickness of the copper layer on the surface after filling needs to be controlled to reduce the difficulty of the subsequent planarization process. TSV hole filling is the key and core step of TSV technology and is also one of the determinants of TSV integration cost, accounting for 26% to 40% of the total production cost. TSV through-hole technology also involves wafer thinning, which leads to potential yield and reliability issues. In order to achieve the design goals of multi-function, high reliability, and miniaturization of modules, packaging process capability is an important factor restricting the development of CPO [53,54].

**Figure 17:**
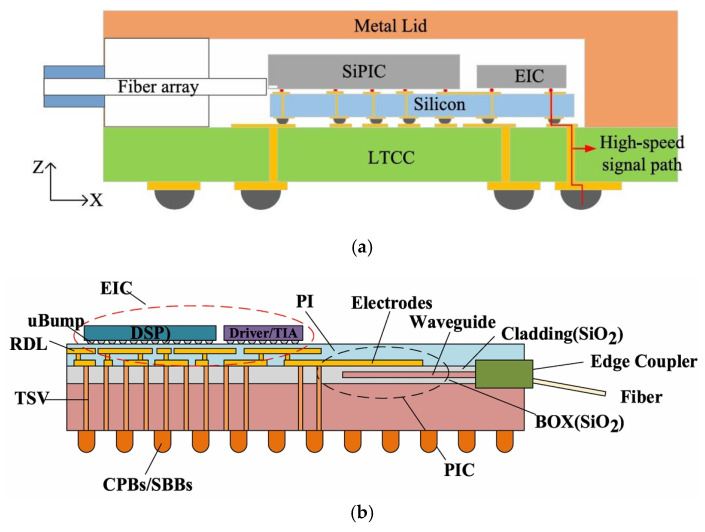
(**a**) Schematic diagram of the 2.5D package solution developed by IMECAS [55]; (**b**) schematic diagram of the 3D package scheme developed by IMECAS [56]. Copyright © 2021, IEEE.

The Institute of Microelectronics of the Chinese Academy of Sciences (IMECAS) has been dedicated to researching advanced packaging technology. IMECAS has developed 2.5D and 3D packaging technologies for CPO module packaging. The 2.5D packaging solution is illustrated in Figure 17a, where the silicon PIC and EIC are flipped onto a 2.5D silicon interlayer and then sub-packaged through a low-temperature ceramic substrate [55]. There are two options for implementing 3D packaging. In the first option, the EIC is placed on the PIC using micro-bumps, and the stacked structure is placed on the PCB through wire connection. In the second option, the PIC is integrated into an active photonic mediator while maintaining passive functionality. Subsequently, based on the EIC, TSV and redistribution layers are prepared, and the EIC is bonded face-to-face with the intermediary. Finally, the substrate and the middle layer are connected by a solder ball, as depicted in Figure 17b in a three-dimensional scheme [56].

### 4.2. Heat Dissipation Technology

Heat causes mechanical stress, which is likely to result in substrate warping and impact the performance of optical coupling and electronic interconnects. The space allocated for optical and electrical components in CPO is very limited, and heat dissipation poses a significant challenge due to the sensitivity of optics to heat. Simulations conducted by the CPO standard working group indicate that under a wind speed of 5 m/s, with the design of 16 CPO modules and 1 switch chip model, the temperature of the switch chip reaches 151.76 °C, rendering normal operation nearly impossible. It is crucial to minimize thermal crosstalk between the ASIC and optics, as well as to address stress and warpage of the overall package. These challenges necessitate innovative cooling solutions, such as advanced heat sinks, liquid cooling, and integrated micro-coolers, to effectively address the thermal issues of CPO modules [57,58].

In recent years, many companies have announced thermal management methods for optoelectronic integration. Intel’s liquid cooling solutions are available for 540 W switching ICs and 56 W per CPO. The results showed that their solution was able to reduce the EIC temperature by 35 °C and the switch chip temperature by 8 °C. Cisco’s heat sink and cold plate assemblies are a very effective solution. Compared to forced air cooling, liquid cooling has a more pronounced cooling effect, such as less noise, more uniform heat dissipation, and does not require an additional power supply to power the fan. In addition, cold plates with thermal interface materials and heat sinks or heat sinks can be used in combination with liquid cooling solutions for better performance [59].

### 4.3. Light Source Integration

The integration of laser sources has always been a difficult point for photonic integrated circuits. Silicon, silicon nitride, and silica are all indirect bandgap materials, which are not easy to emit light and need to be introduced into the light source by other means. There are two ways to place the light source, one is the on-chip integration of the light source and the modulator, which is called “light source internal”, and the other is to package the light source independently, and then couple the light source to the optical path and then couple the modulator, which is called “external light source”.

There are reliability problems in the built-in light source scheme, and the required value of the failure rate needs to be at least an order of magnitude lower than the value that can be provided by the existing technology, which is difficult to achieve. Considering the high reliability of the silicon-based integrated optical path in the absence of a laser, it is suitable for decoupling problematic lasers. The external light source method can reduce the heat of the optical transceiver unit, thereby reducing the power consumption of the optical module, which is the preferred placement method of various manufacturers. There are still some challenges to this technology, including low end-to-end coupling efficiency, increased cost of polarization-maintaining fibers, increased difficulty in optical interconnection engineering, and high-power laser fiber burning.

**Figure 18:**
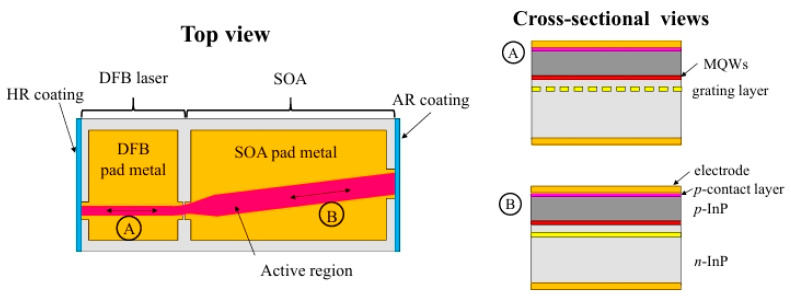
Schematic diagram of a manufactured SOA-integrated DFB laser. The left image is a top view, and the right image is a horizontal screenshot of DFB laser in the A direction and a horizontal screenshot of SOA in the B direction, respectively [61]. Reprinted with permission from © Optical Society of America.

Continuous Wave (CW)-Distributed Feedback Laser (DFB) is currently the best external light source choice for CPO technology. Many researchers have studied the high-power operation capability of O-band DFB lasers. In 2022, Yao’s team reported a high-power CW-DFB laser with a small divergence angle based on the n-type InGaAsP far-field reduction layer design. At 25 °C, the laser power reaches 173 mW@400 mA, the slope efficiency is 0.46 W/A, the working wavelength is in the O-band, the horizontal axis and vertical axis of the far-field divergence angle are less than 20°, the side-mode rejection ratio is greater than 55 dB@300 mA, the Lorentz linewidth is less than 600 kHz, and the Relative Intensity Noise (RIN) is <−155 dB/Hz, which meets the requirements of the CW-WDM MSA multi-source protocol. It can support CPO applications [60]. The semiconductor optical amplifier (SOA) integrated DFB laser structure facilitates the selection of driving conditions to optimize optical power, power conversion efficiency (PCE) and RIN. In 2023, the Daisuke Inoue team introduced a DFB laser with integrated SOA as an external light source for CPO applications. Figure 18 shows a schematic of an SOA integrated DFB laser. DFB lasers with straight mesa stripes and 7° inclined SOA are connected by curved and tapered waveguide sections. The taper length was chosen to suppress higher-order transverse modes. The table width of the SOA section is 7.2 μm. The total length of the device is 2 mm. The device has an optical power of more than 500 mW at 25 °C. By selecting the DFB laser and SOA drive current, an optical power of 350 mW was obtained at 45 °C, with a PCE greater than 25% and an average RIN below −155 dB/Hz [61].

### 4.4. Coupling Structure

Achieving optical coupling of low-loss silicon-based PIC chips in a manufacturable manner has been challenging due to the large pattern mismatch between silicon waveguides and optical fibers. There are generally two coupling methods, surface normal coupling using a grating coupler and edge couplers using a spot size converter (SSC) [62]. Grating couplers (GCs) are typically fabricated by a simple two-step etching process, resulting in vertical optical coupling. Grating couplers with proper taper and apodization designs can provide a pattern size output that matches that of a common single-mode fiber (SMF). However, they often have some drawbacks, including limited bandwidth, center wavelength susceptibility to manufacturing tolerances, ambient temperature variations, and strong polarization dependence. In addition, due to the imperfect mode distribution of GCs and the diffraction losses, it is difficult to achieve coupling losses of less than 1 dB, even with advanced 65 nm CMOS processes. Edge couplers enable small coupling losses and large optical bandwidth, which is ideal for practical applications. However, edge couplers require undercut and deep etching processes during the manufacturing process, resulting in device stability and reliability issues.

**Figure 19:**
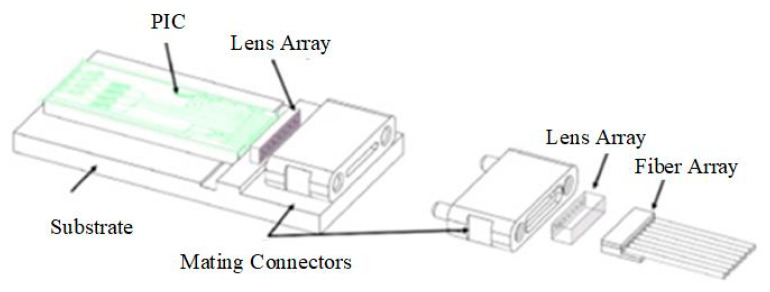
Conceptual view of the detachable PIC/fiber coupling interface [1].

In 2023, the National Laboratory of Solid State Microstructures and the Collaborative Innovation Center for Advanced Microstructures and the Key Laboratory of Microwave Photonics Technology of the College of Engineering and Applied Science of Nanjing University developed a detachable connector interface based on extended beam coupling for edge emission/coupling photonic devices and components [1], as shown in Figure 19. The interface consists of a microlens array and a positioning connector with pin/pinhole positioning on the edge of a transmit PIC or the device side for waveguide output collimation and output beam registration as a permanent section, respectively, and a fiber collimator array with mating connectors on the fiber side as a detachable section. The interface supports multiple parallel ports and can be used for low-loss optical coupling of SiP PICS, III/V lasers, and fiber arrays. This detachable coupling interface achieves coupling losses of less than 1 dB, has the potential to support multiple parallel ports at high density, and is fully weld flow compatible, making it a promising optical I/O solution for CPO applications.

**Figure 20:**
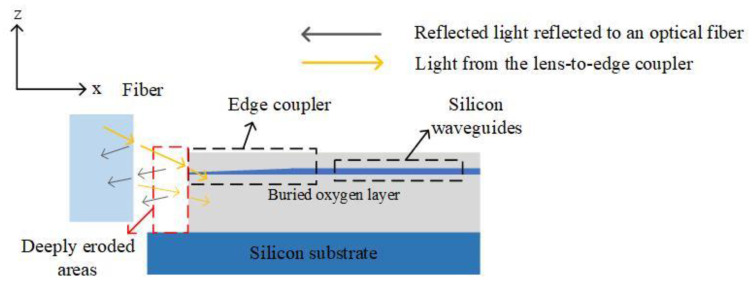
Side view of the fiber end face and the edge coupler forming an FP cavity, and the light reflection occurs on the fiber end face and the edge coupler end face [63].

In the packaging process of commercial silicon photonic chips, the large-scale alignment of optical fibers and silicon photonic chips puts forward higher requirements for the alignment efficiency of optical fibers and edge couplers. In 2023, a study by the Key Laboratory of Special Optical Fiber and Optical Access Networks at Shanghai University showed that efficient alignment of optical fibers and edge couplers can be achieved using a Fabry–Perot (FP) cavity based on the formation between the fiber end face and the edge coupler end face [63]. As shown in Figure 20, an FP cavity is formed between the end face of the fiber and the end face of the edge coupler, which can realize the optimization of the alignment process between the fiber and the edge coupler, and only a single position adjustment of the fiber can achieve high-precision alignment with the edge coupler. The distance between the fiber and the edge coupler and the displacement of the fiber are measured in the sub-micron range in the axial direction (expressed as the x-axis direction) in the optical fiber axial direction (expressed as the x-axis direction). With the scheme proposed by the team, the coupling loss between the fiber and the edge coupler can be controlled to 3.52 dB, which is only 0.22 dB off the standard reference value.

### 4.5. Standardization

On the road to promote the evolution of high-speed communication miniaturization, high-density integration, and high-capacity technologies to the next generation, the traditional architecture is gradually weak, and the formulation of CPO standards can promote the overall upgrading of the industry and the restructuring of the ecological supply chain. By unifying technical standards, the global upstream and downstream industrial chains can be connected. Only through standardization can the products be used in a larger market and meet the needs of more consumers. Standardization can also eliminate technical barriers, promote international economic and trade development and technical exchanges and cooperation, and connect the global upstream and downstream industrial chains through unified technical standards. At present, CPO has a variety of application scenarios, different application scenarios have different requirements, standardization needs to meet the requirements of multiple scenarios, and the diversity of optical engine architecture and technology makes standardization challenging.

The development of CPO requires the coordinated promotion of the industrial chain, which will test the long-term comprehensive strength of optical module manufacturers. The technical route optimization of CPO is essentially the optimization of the entire network architecture, which requires the coordinated promotion of the entire data center industry chain. Among them, on the basis of the existing optical module industry chain, some links also need the participation of exchange chip and equipment manufacturers and component manufacturers. The success of CPOs depends on effective collaboration between optical suppliers, module vendors, and data center operators to align efforts to address user needs, technical feasibility, and economic feasibility.

## 5. Conclusions

Due to its high speed, huge bandwidth, and low power consumption, CPO has become an essential technology in the optical communication industry. Currently, CPO is predominantly employed in Ethernet networks within data centers. Nevertheless, due to the swift progress of artificial intelligence and big data, the implementation of CPO is anticipated to expand into diverse domains. Currently, 2.5D and 3D packaging are the most promising methods for accomplishing high-speed optoelectronic co-packing. The parasitic parameters of the electrical inter-connection between the EIC and PIC in the 3D package are further minimized compared to the 2.5D package. This reduction is advantageous for obtaining increased bandwidth signal connectivity, which is the current emphasis point and trend in CPO technology research. However, there are still several crucial technical challenges that require immediate attention, such as packaging procedures, heat dissipation technology, light source integration, coupling, and so on. Furthermore, the standardization procedure of CPO technology requires prompt improvement. These limitations hinder the progress and advancement of CPO technology, but they also provide chances for innovation and significant advancements. In the future, it is crucial for the industry to cooperate and investigate in order to develop a comprehensive ecosystem for CPO technology, industry, and standards.

## Figures and Tables

**Table 1:** Comparison of CPO results based on silicon photonics integration technology.

Company	Light Source	Optical Connection	Electrical Socket	Soldered	Energy Consumption (PJ/bit)
AMD (Santa Clara, CA, USA)/Ranovus (Ottawa, ON, Canada)	Integrated or remote		√		5
Broadcom (Irvine, CA, USA)	Remote	√		√	5
Cisco(San Jose, CA, USA)	Remote		√		5
Intel (Santa Clara, CA, USA)	Integrated	√	√	√	1.3
Marvell (Silicon Valley, CA, USA)	Integrated		√		5

**Table 2:** Comparison of VCSEL-based CPO results.

	IBM	HPE	Fujitsu	Furukawa
Electrical Interface	16 × 56 Gbps NRZ	4 × 112 Gbps PAM4	16 × 25 Gbps NRZ	8 × 56 Gbps PAM4
IC technology	55 nm BiCMOS	NA	NA	NA
Wavelength/nm	940	990/1015/1040/1065	1060	1060
Laminate interface	Solder or LGA	NA	LGA	LGA
Footprint/${\mathrm{mm}}^{3}$	13 × 13 × 6	NA	7.8 × 16 × 8.0	15.9 × 7.7 × 7.95
Data rate/Gbps	800	400	400	400
Energy Consumption(pJ/bit)	4.2	NA	NA	NA
Bidirectional Bandwidth Density/(Gbps/${\mathrm{mm}}^{2}$)	10.60	NA	6.41	7.32
